# Supplementation with the Traditional Thai Polyherbal Medicine NawaTab Ameliorates Lipid Profiles in High-Fat Diet-Induced Hyperlipidemic Rats

**DOI:** 10.1155/2022/8574756

**Published:** 2022-11-21

**Authors:** Apichaya Niyomchan, Wasapon Chatgat, Bodin Chatawatee, Thaweeporn Keereekoch, Patcharawalai Jaisamut, Sasitorn Chusri, Nongluk Kunworarath

**Affiliations:** ^1^Department of Anatomy, Faculty of Medicine Siriraj Hospital, Mahidol University, Bangkok 10700, Thailand; ^2^Faculty of Traditional Thai Medicine, Prince of Songkla University, Hat Yai, Songkhla 90110, Thailand; ^3^Traditional Thai Medical Hospital, Faculty of Traditional Thai Medicine, Prince of Songkla University, Hat Yai, Songkhla 90110, Thailand; ^4^Traditional Thai Medical Research and Innovation Center, Faculty of Traditional Thai Medicine, Prince of Songkla University, Hat Yai, Songkhla 90110, Thailand; ^5^School of Health Science, Mae Fah Luang University, Muang, Chiang Rai 57100, Thailand

## Abstract

A tablet formulation developed from Nawametho decoction (NawaTab), a traditional Thai herbal mixture described in the Worayokasan scripture, has been used clinically for several years in the management of borderline hyperlipidemic individuals. Nevertheless, scientific evidence supporting its claims has never been identified. This study aimed to describe the antihyperlipidemic properties of NawaTab in a well-described model of high-fat diet (HFD)-induced hyperlipidemic rats. Decoction of Nawametho containing equal quantities of *Aegle marmelos* (L.), *Carthamus tinctorius* L., *Hibiscus sabdariffa* Linn., *Phyllanthus emblica* L., *Piper longum* L., *Piper nigrum* L., *Terminalia bellirica* (Gaertn.) Roxb., *Terminalia chebula* Retz., and *Zingiber officinale* Roscoe were prepared. The HFD-fed rats were administered NawaTab for 4 consecutive weeks starting from the ninth week of HFD treatment at a dose of 125 mg/kg (p.o.). Anthropometric parameters, serum lipid profiles, lipase activity, and liver and renal functional markers were measured. Histopathological examination of the liver and any steatosis was carried out at the end of this study. Consecutive treatment with NawaTab (125 mg/kg/day) in HFD-induced hyperlipidemic rats caused a significant reduction in serum total cholesterol, triglyceride, low-density lipoprotein cholesterol, and very low-density lipoprotein cholesterol levels. However, at the tested dose, NawaTab failed to prevent the onset of hepatic steatosis and adipose tissue accumulation. No adverse events due to the consumption of NawaTab on liver and kidney function markers were noted. These findings are the first suggestive evidence for the lipid-lowering capability of NawaTab. However, to promote the use of this formulation in the management of borderline hyperlipidemic patients, elucidation of the underlying mechanisms of action, quantification of biological markers, and clinical trials of NawaTab are urgently needed.

## 1. Introduction

Cardiovascular diseases (CVDs) are the leading cause of mortality and contribute substantially to decreased quality of life worldwide, particularly in low- and middle-income countries [1–3]. According to a recent systematic review, CVD caused approximately 17.8 million deaths worldwide in 2017, an increase of 21% since 2007 [1]. Moreover, ischemic heart disease and stroke were ranked as the first and second causes of disability-adjusted life-years in both the 50–74 years and ≥75 years age groups in 2019 [2]. As a major preventable risk factor for CVD, the constantly increasing incidence of hyperlipidemia in several countries has become the main threat to public health [3–5].

Statins successfully reduce total cholesterol (TC) and low-density lipoprotein cholesterol (LDL-C) levels in patients with elevated LDL-C, thereby reducing the risk of cardiovascular development and mortality. The administration of statins was found to be associated with elevated liver enzyme transaminases, increased blood glucose and glycated hemoglobin levels, and the occurrence of muscle-related symptoms [6]. Nonadherence to this medication was also noted as a limitation of statins and has been associated with an increased risk of CVD and mortality. Studies have revealed that only half of the patients continue statin therapy through the first year, and discontinuation rates were approximately 75% in the second year [6]. Therefore, effective treatment of hyperlipidemia with fewer side effects is urgently required to control raised LDL-C levels and thereby limit the morbidity and mortality of CVD.

For many decades, medicinal plants and polyherbal formulations have been used throughout the world for their hypolipidemic activity [7, 8]. These antihyperlipidemic effects, particularly those of individual medicinal plants, have been tested using both animal and clinical studies. In contrast, only limited information has been established regarding polyherbal formulations/mixtures, which are regularly utilized in several traditional medical systems. Some traditional Chinese medicinal formulas with hypolipidemic effects have been reported, such as Danggui Buxue decoction supplementation of diabetic atherosclerosis rats and hyperlipidemic model rats [9] and Shengmai-san [10], which resulted in a remarkable decrease in TC and LDL-C levels. Furthermore, treating postmenopausal women with hypercholesterolemia with the Danshen–Gegen formula resulted in a significant improvement in intima-media thickness and a considerable reduction in TC and LDL-C levels [11]. Based on these earlier findings [9–11], it is interesting to note that traditional polyherbal medicines can be valuable and promising sources for antihyperlipidemic drug discovery in addition to the utilization of single herbs or plant-derived compounds.

The current experiment was thus designed to prove whether a tablet formulation of a polyherbal formula, Nawametho decoction, exhibits an antihyperlipidemic effect in a model of HFD-fed rats. This herbal medicine is described in the Worayokasan scripture and is regularly employed in managing borderline hyperlipidemic patients at Bantakhun Hospital (Surat Thani, Thailand). The formulation consists of equal quantities of nine medicinal plants, including *Aegle marmelos* (L.), *Carthamus tinctorius* L., *Hibiscus sabdariffa* Linn., *Phyllanthus emblica* L., *Piper longum* L., *Piper nigrum* L., *Terminalia bellirica* (Gaertn.) Roxb., *Terminalia chebula* Retz., and *Zingiber officinale* Roscoe. These components of Nawametho are commonly used in traditional medicine in many countries, even in Thailand. They have been used to treat different ailments including hyperlipidemia, digestive disorder, liver, and cardiovascular diseases, to help fat burning, to reduce weight, and to improve blood circulation [12–23]. Although Nawametho decoction has been used clinically for many years, and the antihyperlipidemic effects of each of these herbal components have been documented, this polyherbal decoction has never been scientifically examined.

## 2. Materials and Methods

### 2.1. Collection of Plant Materials and Preparation of NawaTab

Nine well-known medicinal plants, as noted in Table 1, were provided by a licensed local pharmacy (Farshen Orsot Part., Ltd., Phatthalung, Thailand) and then authenticated and verified by a traditional Thai botanist, Assistant Professor Dr. Katesarin Maneenoon. Botanical reference materials of the medicinal plants were assigned and preserved in Materia Medica at the Faculty of Traditional Thai Medicine, Prince of Songkla University (Songkhla, Thailand). The cleaned plant parts were oven-dried at 80°C for 48 h, powdered, and passed through 40 meshes. These ground plant parts were separately stored in airtight containers, and the same batch was applied for the entire study to prevent batch-to-batch variation.

Nawametho decoction was made according to the traditional procedure used in Thailand as follows: the formulation (100 g), which contained an equal amount of each powdered plant part in a fine muslin container (30 cm^2^) mixed with 1.5 liters of distilled water, was boiled for 2 h at 98 ± 3°C. This decoction was filtered, cooled at room temperature overnight, and subjected to spray drying to obtain a dark brown-colored powder with an extraction yield of 10.33% (w/w; dry weight basis). Liquid chromatography-mass spectrometry (LC-MS) analysis was performed to identify the chemical compositions of Nawametho decoction. The result from the LC-MS analysis is shown in Supplementary information S1. NawaTab, which contains 385 mg of Nawametho decoction per tablet, was prepared as described in petty patent number 1903002586.

### 2.2. Experimental Animal Husbandry and Ethics Committee Approval

Four-week-old male Wistar rats weighing 100–120 g were ordered from Nomura Siam International Co, Ltd. (Bangkok, Thailand). The rats were housed in stainless steel wire mesh cages under controlled environmental conditions consisting of a relative humidity of 55 ± 10%, a temperature of 22 ± 2°C, and a 12 h light/dark regime at the Southern Laboratory Animal Facility (Prince of Songkla University). The animals were provided with free access to an irradiation-sterilized standard diet (No. CP 082, Perfect Companion Group Co., Ltd., Bangkok, Thailand) and filtered water *ad libitum*. The animals were acclimatized to the laboratory conditions for one week prior to the experiments. All possible attempts were made to reduce animal cruelty and decrease the number of animals used. The Animals Ethical Committee of Prince of Songkla University (MOE 0521.11/876) approved the study protocols.

### 2.3. Induction of Hyperlipidemia in Rats and the Experimental Design

Hyperlipidemia in rats was induced by feeding with a HFD (58 V8 TestDiet®, Singapore) with an energy of 4.6 kcal/g, comprising 46.1% calories from fat, 18.1% from protein, and 35.8% from carbohydrate. The standard diet used in this present work contained 25.5% protein, 5.7% fat, 2.1% fiber, 6.33% water, vitamins, and minerals and yielded 4.02 kcal/g and 1.77% total energy from fat. Twenty-four healthy Wistar rats were randomly assigned into four groups after a one-week quarantine and acclimatization period. The detailed study groups were as follows: Group 1 (the control group) received standard palletized diets and distilled water (vehicle) throughout the experiment. Groups 2–4 were fed a customized HFD for 12 weeks. Rats in Groups 2, 3, and 4 were orally administered vehicle (Group 2), NawaTab (125 mg/kg) (Group 3), or simvastatin (a standard hypolipidemic drug; 10 mg/kg) (Group 4) for 4 consecutive weeks starting from the ninth week of HFD treatment [24, 25].

### 2.4. Food Consumption and Anthropometric Assessments

Food intake was assessed once a day as the difference between the amount of food before refilling and the food remaining on the next day and expressed as g/head/day. Body weight was taken daily, while the waist and chest circumferences were recorded every four weeks for the last 12 weeks. Body weight gain was calculated from the difference between final body weight and initial body weight. The food efficiency ratio (FER) was evaluated based on the weight gain of the rat (g/day) divided by its intake of the food (g/day).

### 2.5. Analysis of Fecal Lipids

The feces of the rats were collected daily and oven-dried at 45–50°C for 24 h. The samples were then pulverized and kept at −20°C for analysis of the total lipids. As previously described with slight modifications [26], 3 g of the feces were extracted with 6 mL of a mixture of chloroform and methanol (1 : 1, v/v) and continuously stirred using an incubator shaker for 2 h. The resultant extract was collected by centrifugation at 3000 rpm for 30 min. The supernatant (5 mL) was taken and oven-dried to obtain a constant weight of the amount of lipids.

### 2.6. Measurements of Relative Organ Weight, Adipose Tissues, and Hematological and Biochemical Parameters

After the 12-week treatment, the rats were fasted overnight and fully anesthetized with 100 mg/kg thiopental sodium (Scott-Edil Pharmacia Ltd., Chandigarh, India). Blood samples were taken directly via cardiac puncture and processed for biochemical and hematological assessments in ethylenediaminetetraacetic acid (EDTA)-coated test tubes and nonheparinized tubes, respectively. All biochemical and hematological parameters were analyzed by National Healthcare Systems Co., Ltd. (N Health Hat-Yai, Songkhla, Thailand) according to standard procedures as described below.

The serum was separated by centrifugation at 3000 rpm for 10 min. Assessment of lipase activity and the levels of TC, triglyceride (TG), high-density lipoprotein cholesterol (HDL-C), LDL-C, and very low-density lipoprotein cholesterol (VLDL-C) were determined using a Cobas® 6000 analyzer (Roche Diagnostics, Penzburg, Germany). Biomarkers of renal and liver function, including levels of alkaline phosphatase (ALP), alanine aminotransferase (ALT), aspartate aminotransferase (AST), blood urea nitrogen (BUN), creatinine, total bilirubin, direct bilirubin, total protein, globulin, and albumin, were also analyzed.

Blood samples with EDTA were used to evaluate hematological parameters, including the white blood cell count (neutrophil, lymphocyte, monocyte, eosinophil, and basophil counts), red blood cell count, hemoglobin, hematocrit, platelet count, mean corpuscular volume, mean corpuscular hemoglobin, mean corpuscular hemoglobin concentration, and red cell distribution width, using an ADVA 2120i hematology system (Siemens Healthineers AG, Erlangen, Germany).

At the end of the experiment, internal organs (heart, liver, spleen, and kidney) and brown, subcutaneous, and visceral (epididymal, retroperitoneal, and mesenteric) adipose tissues were carefully dissected, and their relative weights were calculated. The liver was examined for any gross lesions and kept in 10% buffered formaldehyde solution for fixation preparatory to histopathological examination.

### 2.7. Histopathological Evaluation of Hepatic Steatosis

Liver tissues were fixed in 10% neutral buffered formalin for 24 h, dehydrated with alcohol, and embedded in paraffin wax. Serial sections were cut by a rotatory microtome (Leica RM2035, Nussloch, Germany) with 6 *μ*m thickness. After deparaffinization, the sections were stained with hematoxylin-eosin (H&E). Microscopic examinations were conducted at magnifications of 20× using an Olympus Bx53F2 microscope and digital camera attached to CellSens Standard Software (Olympus Optical, Co. Ltd, Tokyo, Japan), which was used to calculate the percentage of hepatocytes containing lipid droplets per total hepatocyte within the hepatic area of 4.5 × 10^12^ mm^2^. Hepatocellular steatosis was categorized as follows: grade 0, 1, 2, and 3 if steatosis occupied less than 5%, 5–33%, 34–66%, and more than 67% of the hepatic parenchyma, respectively.

### 2.8. Statistical Analysis

The data are presented as the mean ± the standard error of the mean and analyzed by one-way analysis of variance (ANOVA) using SPSS version 17 software (SPSS Inc., Chicago, IL) followed by Tukey's post hoc test. Differences were considered statistically significant at *p* < 0.05.

## 3. Results

### 3.1. Effect of NawaTab on the Food Consumption, Body Weight, and FER of HFD-Induced Hyperlipidemic Rats

**Figure 1:**
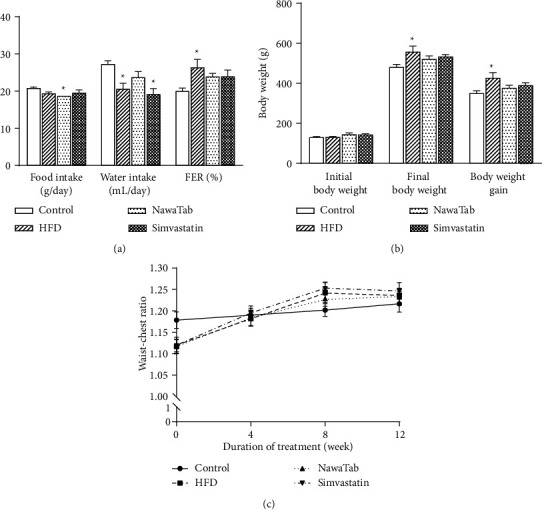
Effect of NawaTab on food intake, water intake, FER (a), body weight (b), and waist-chest ratio (c) of high-fat diet (HFD)-induced hyperlipidemic rats. Data are expressed as mean ± S.E.M., *n* = 6 animals/group. ^*∗*^*p* < 0.05, significantly different from the control group.

Based on earlier reports, as shown in Table 1, and the traditional utilization of NawaTab as a hypolipidemic agent, the effects of this herbal formula supplementation and HFD consumption on the body weights and fat accumulation of the experimental rats are reported in Figure 1. During the 12-week experiment, it was found that the rats exposed to the HFD did not have a significantly different food intake than controls, but had a significantly lower intake of water. As expected, FERs, body weights, and body weight gain were significantly higher in the HFD group than in the control group. Supplementation with either NawaTab or simvastatin caused a slight reduction in the FERs, body weights, and body weight gain of the animals fed a HFD. Furthermore, NawaTab at a dose of 125 mg/kg/day significantly decreased the food consumption of these animals. The rats exposed to either the standard control diet or HFD did not show significant changes in their waist-chest ratio.

### 3.2. NawaTab Supplementation Improves the Lipid Profile of HFD-Induced Hyperlipidemic Rats

Except for fecal lipid contents, the activities of lipase and the levels of TC, TG, HDL-C, LDL-C, and VLDL-C of the HFD-fed group were higher than those of the rats fed the standard diet, indicating that the animal hyperlipidemia model that was developed was accurate (Table 2). Supplementation of the hyperlipidemic rats with this herbal formula at a dose of 125 mg/kg/day for 4 weeks significantly reduced serum TC, TG, LDL-C, and VLDL-C levels. It should be further noted that serum TG and VLDL-C levels of rats in the HFD group receiving NawaTab were similar to those of the negative control group. However, treating the hyperlipidemic animals with this polyherbal decoction did not cause serum HDL-C changes. Although reductions in serum TC, TG, HDL-C, LDL-C, and VLDL-C levels in HFD-fed animals were observed after treatment with simvastatin at a dose of 10 mg/kg/day for 4 weeks, only the levels of TC, HDL-C, and LDL-C were found to be significant. In the present study, there were no significant changes in the fecal lipid content or the lipase activity of the hyperlipidemic rats supplemented with either NawaTab or simvastatin.

The results illustrated in Table 2 show that feeding animals a HFD caused a significant elevation in the accumulation of visceral and subcutaneous white adipose tissues compared to the negative control group, which received the standard diet. Oral administration of either NawaTab or simvastatin to rats fed a HFD did not significantly reduce adipose tissue accumulation. A slight reduction in the amount of retroperitoneal, mesenteric, and subcutaneous adipose tissues was found in the HFD-fed group treated with this herbal decoction. In the hyperlipidemic rats administered simvastatin, a small decrease in the amount of mesenteric and subcutaneous adipose tissues was observed. It has been well-documented that brown adipose tissue plays an important role in thermoregulation, body weight regulation, glucose, and lipid homeostasis [27]. However, treatment with either NawaTab or simvastatin in rats fed a HFD did not affect the level of brown adipose tissue accumulation.

### 3.3. The Administration of NawaTab Did Not Protect against HFD-Induced Liver Steatosis in Rats

Feeding a HFD for 12 weeks significantly induced liver steatosis in rats, by 33-fold compared to the negative control group, as presented in Table 2. Compared to the vehicle-treated group, NawaTab-treated HFD-induced liver steatosis rats did not experience a significant change in the percentage of hepatocytes containing lipid droplets or the grading of hepatic steatosis. Conversely, simvastatin administration appeared to significantly reduce the severity of HFD-induced liver steatosis, from grade 3 (92.4 ± 3.9%) to grade 1 (13.0 ± 2.8%).

**Figure 2:**
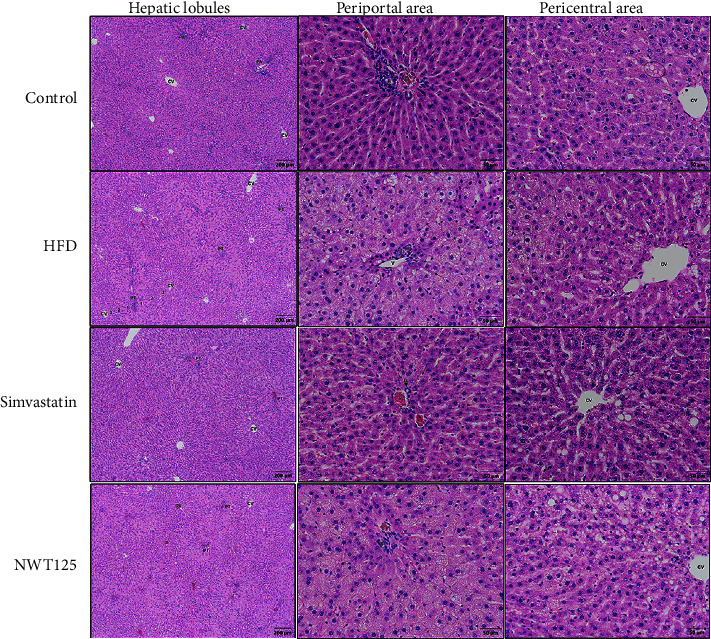
Microscopic images of liver tissues stained with hematoxylin and eosin (200×) of normal Wistar rats (Control) and high-fat diet (HFD)-induced hyperlipidemic rats. Evidence of hepatic steatosis was reported from HFD-induced hyperlipidemic rats supplemented with vehicle (HFD), 10 mg/kg/day simvastatin, or 125 mg/kg/day NawaTab (NWT125). CV, central vein; PA, portal area; (V) branches of the portal vein; (A) hepatic artery; (B) bile duct; 1, periportal area; 2, midlobular region; 3, pericentral zone.

Pathological images of liver tissue stained with H&E revealed abundant cytosolic lipid droplets in the hepatocytes and both macro- and microvesicular steatosis in the HFD group (Figure 2). As expected, treatment with simvastatin exhibited significant efficacy in defense against these pathological features. However, the consumption of NawaTab at a dose of 125 mg/kg/day for 4 weeks appeared to fail to protect against these changes (Figure 2).

### 3.4. Effect of NawaTab on the Relative Weight of Organ and Biochemical and Hematological Parameters of HFD-Induced Hyperlipidemic Rats

Any undesirable effects of the plant extract on rats' major organs would likely cause alterations in the organ/body ratio, hematological parameters, and biochemical parameters, as illustrated in Table 3. No such changes were noted in this study, which confirms the nontoxicity of NawaTab. No major changes were observed, except in the kidneys and in the weight of the liver, spleen, and heart of HFD-induced rats. The results showed that feeding a HFD significantly reduced the weight of the kidney, whereas treatment with NawaTab and simvastatin did not cause a notable improvement in this organ weight. Furthermore, there were no alterations in any tested biochemical parameters of vehicle-treated, simvastatin-treated, or NawaTab-treated hyperlipidemic rats. In contrast, the percentages of lymphocytes, monocytes, and eosinophils were significantly changed in the vehicle-treated HFD-fed rats relative to the rats in the normal control group. Supplementation with either simvastatin or NawaTab failed to cause an improvement in these hematological parameters.

## 4. Discussion

CVD is one of the most important causes of death in several countries, and the rising number of people with hyperlipidemia as a major risk factor for this disease has become a global concern. In Thailand, six in ten Thai people live with at least one form of dyslipidemia [28]. Analysis has shown that approximately 30%, 47%, and 39% of Thai individuals have high LDL-C, low HDL-C, and high TGs, respectively. Poor public awareness of dyslipidemia should be considered a factor, but a correlation between CVD markers, carotid intima-media thickness, and hyperlipidemia among the Thai population has been demonstrated [29]. Furthermore, previous findings indicated that 27.1% of the Thai worker population and 38.7% of chronic disease patients were self-prescribed herbal medicine for dyslipidemia [30,31]. However, there is limited scientific information to show the efficiency and safety of traditionally used medicines.

Therefore, in the present work, we demonstrated for the first time that oral administration of NawaTab (125 mg/kg/day) for only 4 weeks effectively reduced plasma TC, TG, LDL-C, and VLDL-C levels in HFD-induced hyperlipidemic rats. Medicinal plants have been highlighted as alternative sources of antihyperlipidemic agents. According to an intensive review by Bahmani and his colleagues [7], some well-known medicinal plants, such as *Cynara cardunculus* (artichoke), *Trigonella foenum graecum* L (fenugreek), *Allium sati*vum L (garlic), *Glycine* max (soybean), and *Commiphora mukul* (guggul, gugulipid) were able to alleviate TC, TG, and LDL-C levels in clinical studies. However, it should be noted that their treatment periods were between 6 and 12 weeks.

Several mechanisms involved in the antihyperlipidemic effects of medicinal plants and plant-derived compounds have been established. A pancreatic lipase inhibitor, such as orlistat, leading to a decrease in lipid absorption is an appealing approach for antihyperlipidemic agent discovery. Plant-derived polyphenols have been reported as the most important sources of prospective pancreatic lipase inhibitors to varying degrees [32]. Among these compounds, gallic acid, an active constituent found in *P. emblica*, *T. bellirica*, and *T. chebula,* had remarkable antipancreatic lipase activity [33]. Even though five out of nine herbal components of NawaTab, including *H. sabdariffa* [34], *P. longum* [35], *P. nigrum* [36], *T. bellirica* [37], and *Z. officinale* [38], have been shown to produce antipancreatic lipase activity, treatment of HFD-fed rats with NawaTab did not result in significant changes in their lipase activity or fecal lipid contents in our study. The adipogenesis inhibition mechanisms of simvastatin remain unclear, but this HMG-CoA reductase inhibitor is widely used to lower LDL-C in patients with hyperlipidemia and cardiovascular diseases. With the exception of *T. bellirica*, all herbal components of NawaTab demonstrated an inhibitory effect on adipogenesis in 3T3-L1 preadipocytes [39–46]. These medicinal plants were shown to either suppress lipid and TG accumulation, inhibit the proliferation of adipocytes, or initiate fat cell apoptosis. It is speculated that the hypolipidemic effect obtained from NawaTab is at least involved in the inhibitory properties of its herbal component toward adipogenesis and pancreatic lipase activity. However, further examinations are required to elucidate the lipid-lowering mechanisms of NawaTab.

Similar to the hypolipidemic effects of NawaTab, previous *in vivo* experiments revealed that its individual herbal components also decrease plasma TC, TG, and LDL-C levels. *H. sabdariffa* [47], *P. emblica* [48], *P. longum* [49], *P. nigrum* [50], and *T. chebula* [51] were able to improve HDL-C levels and reduce TC and TG levels in diet-induced hyperlipidemia rat models. *C. tinctorius* [41], *H. sabdariffa* [43], and *T. bellirica* [52] also exhibit hypolipidemic effects in other hyperlipidemic animals, including rabbits, hamsters, and mice. Furthermore, oral administration of *A. marmelos* [53], *P. emblica* [54], *T. bellirica* [37], and *Z. officinale* [55] has been found to improve the lipid profiles of diabetic animal models. Additionally, consecutive administration of NawaTab for 28 days at a dose of 125 mg/kg/day did not cause undesirable changes in organ or body weight, hematological parameters, or kidney and liver function markers. According to polyherbalism theory, the use of multiple medicinal plant combinations can result in a synergistic effect, thereby offering extra therapeutic effectiveness and decreasing unpleasant side effects. The specific active chemical constituents that might be responsible for the lipid-lowering effects of NawaTab, such as aegeline, halfordinol, ethyl ether aegeline, esculetin, hydroxysafflor yellow, delphinidins, digallic acid, gallic acid, piperlonguminine, piperine, pipernonaline, and galanolactone, need to be quantified.

## 5. Conclusion

In conclusion, our findings demonstrated for the first time that NawaTab, which is regularly used for the management of borderline hyperlipidemic patients, exhibits antihyperlipidemic effects. The formulation reduced plasma TG, TC, and LDL-C levels of HFD-induced hyperlipidemic rats at a dose of 125 mg/kg/day for only 4 weeks without adverse events. Although our results provide preliminary support for the traditional utilization of NawaTab, further scientific investigation is required to describe the mechanisms of action, particularly NawaTab's adipogenesis inhibition effect. Moreover, the active agents need to be determined and further applied as quality markers of NawaTab.

## Figures and Tables

**Table 1:** Reported hypolipidemic effects of nine medicinal plants used for the preparation of Nawametho.

Medicinal plants [parts used/voucher specimen No]	Reported hypolipidemic effects [references]	Proposed active constituents [references]
*Aegle marmelos* (L.)	Inhibition of adipogenesis (3T3-L1 cells) [1]	Halfordinol, ethyl ether aegeline, and esculetin [1]
(Fruits/MTM08-01)	(−) TC, TG, FFA; (+) HDL-C (STZ-induced diabetic rats) [2]	Aegeline 2 [2]
*Carthamus tinctorius* L.	Inhibition of adipogenesis (3T3-L1 cells) [3]	Hydroxysafflor yellow A [3]
(Flowers/MTM08-23)	(−) TC, TG, LDL-C; (+) HDL-C (hyperlipidemic mice) [3]	
*Hibiscus sabdariffa* Linn.	Antipancreatic lipase activity (IC_50_∼160–790 mg/mL) [4]	Delphinidins [4]
(Flowers/ARDA18-05)	Inhibition of adipogenesis (3T3-L1 cells) [5]	
	(−) L-TC, L-TG, TC, TG, LDL-C, HDL-C	
	(Hyperlipidemic hamsters [5] and rats [6])	
*Phyllanthus emblica* L.	Inhibition of adipogenesis (3T3-L1 cells) [7]	Digallic acid [8]
(Fruits/MTM08-72)	(−) TC (high-fat-diet-induced hyperlipidemia rats) [8]	
	(−) LDL-C; (+) HDL-C (fructose-fed ovariectomized rats) [9]	
*Piper longum* L.	Antipancreatic lipase activity (IC_50_∼0.175 mg/mL) [10]	Oil [10]
(Flowers/ARDA18-06)	Inhibition of adipogenesis (3T3-L1 cells) [11]	Piperlonguminine [11]
	(−) L-TC, L-TG, TC, TG, LDL-C, (+) HDL-C	Piperlonguminine, piperine, [12] and pipernonaline
	(Cholesterol-rich diet-induced hyperlipidemia rats) [12]	
*Piper nigrum* L.	Antipancreatic lipase activity (0.03 *μ*g/mL orlistat eq.) [13]	
(Fruits/MTM08-78)	Inhibition of adipogenesis (3T3-L1 cells) [14]	Piperine [14]
	(−) body weight, total fat, %fat, TC, TG, FFA,	Piperonal [15]
	(−) LDL-C, (+) HDL-C (HFD-induced obese rats) [15]	
*Terminalia bellirica* (Gaertn.) Roxb.	Antipancreatic lipase activity (65.71 *μ*g/mL) [16]	Gallic acid [16]
(Fruits/MTM08-91)	(−) TC, TG, LDL-C/HDL-C (spontaneously obese type 2 diabetic TSOD mice) [16] (−) TC, TG, phospholipid (cholesterol-rich diet-induced hyperlipidemia rabbits) [17]	
*Terminalia chebula* Retz.	Inhibition of adipogenesis (3T3-L1 cells) [18]	Gallic acid [18]
(Fruits/MTM08-92)	(−) TC, TG, phospholipid (cholesterol-rich diet-induced hyperlipidemia rabbits) [17] (−) TC, TG, (+) HDL-C	
	(Atherogenic diet-induced hyperlipidemic rats) [19]	
*Zingiber officinale* Roscoe.	Antipancreatic lipase activity (65.71 *μ*g/mL) [20]	Galanolactone [22]
(Rhizome/MTM08-98)	Inhibition of adipogenesis (3T3-L1 cells) [21]	
	(−) TC, TG, (+) HDL-C (STZ-induced dyslipidemia diabetic rats) [22]	

TC, total cholesterol; TG, triglycerides; HDL-C, high-density lipoprotein cholesterol; LDL-C, low-density lipoprotein cholesterol; L-TC, liver total cholesterol; L-TG, liver triglycerides; FFA, free fatty acid; STZ, streptozotocin.

**Table 2:** Effect of NawaTab on serum lipid profiles, the relative weight of adipose tissue, hepatic steatosis, fecal lipid excretion, and lipase activity of high-fat diet (HFD)-induced hyperlipidemic rats.

Parameters^*∗*^	Control	HFD		
		Vehicle	NawaTab	Simvastatin
			(125 mg/kg/day)	(10 mg/kg/day)
TC (mg/dL)	57.3 ± 2.8^a^	105.7 ± 9.4^b^	79.8 ± 3.3^c^	82.2 ± 2.8^c^
TG (mg/dL)	112.0 ± 13.1^a,b^	149.0 ± 19.2^b^	77.7 ± 12.7^a^	109.2 ± 12.1^a,b^
HDL-C (mg/dL)	22.3 ± 1.4^a^	29.0 ± 1.9^b^	24.5 ± 0.7^a,b^	23.8 ± 0.6^a^
LDL-C (mg/dL)	3.3 ± 0.3^a^	8.0 ± 0.8^b^	5.8 ± 0.3^c^	6.0 ± 0.4^c^
VLDL-C (mg/dL)	22.3 ± 2.6^a,b^	29.7 ± 3.9^b^	15.5 ± 2.5^a^	21.8 ± 2.4^a,b^
Relative weight of adipose tissues (%)				
(i) Retroperitoneal adipose tissue	3.64 ± 0.13^a^	5.23 ± 0.21^b^	5.04 ± 0.28^b^	5.40 ± 0.30^b^
(ii) Mesenteric adipose tissue	2.36 ± 0.07^a^	3.66 ± 0.14^b^	3.17 ± 0.17^b^	3.35 ± 0.29^b^
(iii) Epididymal adipose tissue	2.62 ± 0.12^a^	3.99 ± 0.18^b^	4.09 ± 0.25^b^	4.20 ± 0.27^b^
(iv) Subcutaneous adipose tissue	2.40 ± 0.17^a^	3.84 ± 0.36^b^	3.11 ± 0.21^a,b^	3.66 ± 0.40^b^
(v) Brown adipose tissue	0.07 ± 0.01^a^	0.08 ± 0.01^a,b^	0.11 ± 0.01^b^	0.11 ± 0.01^b^
Hepatic steatosis (%)	2.8 ± 1.3^a^	92.4 ± 3.9^b^	86.4 ± 4.0^b^	13.0 ± 2.8^c^
Fecal lipid (mg/g)	66.27 ± 2.87^a^	51.00 ± 1.34^b^	42.40 ± 0.51^b^	45.00 ± 3.85^b^
Lipase (U/L)	4.00 ± 0.63^a^	9.67 ± 1.12^b^	8.83 ± 1.74^b^	6.00 ± 0.68^a,b^

The results are given as means ± SEM (*n* = 6). Values in the same row sharing a common superscript are not significantly different (*p* < 0.05). TC, total cholesterol; TG, triglyceride; HDL-C, high-density lipoprotein cholesterol; LDL-C, low-density lipoprotein cholesterol; VLDL-C, very low-density lipoprotein cholesterol.

**Table 3:** Effect of NawaTab on the relative weight of organs and biochemical and hematological parameters of high-fat diet (HFD)-induced hyperlipidemic rats.

Parameters^*∗*^	Control	HFD		
		Vehicle	NawaTab	Simvastatin
			(125 mg/kg/day)	(10 mg/kg/day)
Relative weight of organs (%)				
Heart	0.24 ± 0.01^a^	0.22 ± 0.01^a^	0.22 ± 0.01^a^	0.22 ± 0.00^a^
Liver	2.75 ± 0.13^a^	2.62 ± 0.08^a^	2.47 ± 0.07^a^	2.48 ± 0.08^a^
Kidney	0.56 ± 0.02^a^	0.45 ± 0.01^b^	0.49 ± 0.01^b^	0.48 ± 0.01^b^
Spleen	0.16 ± 0.00^a^	0.14 ± 0.01^a^	0.16 ± 0.01^a^	0.14 ± 0.01^a^
Biochemical parameters				
Alkaline phosphatase (U/L)	70.5 ± 5.4^a^	87.3 ± 5.8^a^	80.0 ± 7.9^a^	82.7 ± 7.8^a^
Alanine aminotransferase (U/L)	33.0 ± 3.5^a^	41.0 ± 8.4^a^	35.0 ± 3.5^a^	30.8 ± 2.7^a^
Aspartate aminotransferase (U/L)	103.0 ± 10.1^a^	126.2 ± 7.5^a^	124.0 ± 4.1^a^	131.2 ± 17.2^a^
Total bilirubin (mg/dL)	0.15 ± 0.0^a^	0.20 ± 0.00^a^	0.22 ± 0.02^a^	0.20 ± 0.00^a^
Direct bilirubin (mg/dL)	0.10 ± 0.00^a^	0.10 ± 0.00^a^	0.10 ± 0.00^a^	<0.10^a^
Total protein (g/dL)	5.70 ± 0.07^a^	5.93 ± 0.08^a^	5.80 ± 0.11^a^	5.77 ± 0.08^a^
Globulin (g/dL)	2.90 ± 0.05^a^	2.95 ± 0.03^a^	2.90 ± 0.08^a^	2.88 ± 0.05^a^
Albumin (g/dL)	2.80 ± 0.03^a^	2.98 ± 0.22^a^	2.90 ± 0.07^a^	2.88 ± 0.13^a^
Blood urea nitrogen (mg/dL)	19.5 ± 0.5^a^	17.8 ± 0.5^a^	16.3 ± 0.9^a^	17.8 ± 1.1^a^
Creatinine (mg/dL)	0.46 ± 0.06^a^	0.39 ± 0.02^a^	0.41 ± 0.02^a^	0.34 ± 0.01^a^
Hematological parameters				
WBC (10^3^/mL)	3.18 ± 0.24^a^	3.49 ± 0.44^a^	4.43 ± 0.73^a^	3.78 ± 0.69^a^
Neutrophils (%)	7.52 ± 1.36^a^	3.48 ± 0.53^a^	7.47 ± 2.37^a^	9.20 ± 2.17^a^
Lymphocytes (%)	54.00 ± 6.24^a^	76.58 ± 2.64^b^	78.03 ± 1.55^b^	78.25 ± 2.33^b^
Monocytes (%)	35.27 ± 5.81^a^	17.52 ± 2.16^b^	13.30 ± 1.53^b^	10.98 ± 1.31^b^
Eosinophils (%)	2.68 ± 0.34^a^	2.13 ± 0.54^a,b^	0.95 ± 0.37^b^	1.55 ± 0.42^a,b^
Basophils (%)	0.53 ± 0.11^a^	0.28 ± 0.05^a,b^	0.25 ± 0.18^a,b^	0.02 ± 0.02^b^
RBC (10^6^/mL)	8.74 ± 0.12^a^	8.26 ± 0.20^a^	8.37 ± 0.21^a^	8.62 ± 0.16^a^
Hemoglobin (g/dL)	15.58 ± 0.32^a^	15.00 ± 0.40^a^	15.47 ± 0.26^a^	15.27 ± 0.27^a^
Hematocrit (%)	48.83 ± 0.98^a^	46.45 ± 1.45^a^	47.60 ± 0.83^a^	47.60 ± 0.69^a^
Platelet count (10^3^/mL)	719.5 ± 30.6^a^	660.3 ± 22.8^a^	683.2 ± 25.4^a^	644.8 ± 24.9^a^
MCV (fL)	55.83 ± 0.43^a^	56.20 ± 0.51^a^	56.93 ± 0.62^a^	55.30 ± 0.68^a^
MCH (pg)	17.82 ± 0.15^a^	18.17 ± 0.11^a,b^	18.50 ± 0.18^b^	17.73 ± 0.23^a^
MCHC (g/dL)	31.92 ± 0.20^a^	32.32 ± 0.17^a^	32.50 ± 0.19^a^	32.08 ± 0.23^a^
RDW (%)	12.55 ± 0.21^a^	12.57 ± 0.37^a^	12.38 ± 0.15^a^	12.70 ± 0.16^a^

The results are given as means ± SEM (*n* = 6). Values in the same row sharing a common superscript are not significantly different (*p* < 0.05). WBC, white blood cells; RBC, red blood cells; MCV, mean corpuscular volume; MCH, mean corpuscular hemoglobin; MCHC, mean corpuscular hemoglobin concentration; RDW, red cell distribution width.

## Data Availability

The data used to support the findings of this study are available from the corresponding author upon request.

## Abbreviations

ALT: Alanine aminotransferase
ALP: Alkaline phosphatase
BUN: Blood urea nitrogen
CVD: Cardiovascular diseases
HFD: High-fat diet
HDL-C: High-density lipoprotein cholesterol
LDL-C: Low-density lipoprotein cholesterol
MCH: Mean corpuscular hemoglobin
MCHC: Mean corpuscular hemoglobin concentration
MCV: Mean corpuscular volume
NawaTab: A tablet formulation containing Nawametho decoction
RBC: Red blood cell count
RDW: Red cell distribution width
TC: Total cholesterol
TG: Triglyceride
VLDL-C: Very low-density lipoprotein cholesterol
WBC: White blood cell count.

## Supplementary Materials

Supplementary Materials
*Supplementary Information 1*. Chemical compositions of Nawametho decoction.
