# Supplementary material for: Supplementation with the Traditional Thai Polyherbal Medicine NawaTab Ameliorates Lipid Profiles in High-Fat Diet-Induced Hyperlipidemic Rats
Source: Evid Based Complement Alternat Med. 2022 Nov 21;2022:8574756. doi: 10.1155/2022/8574756 (PMC9705105; doi:10.1155/2022/8574756)
Supplement: Supplementary Materials — Supplementary Information 1. Chemical compositions of Nawametho decoction. [file 8574756.f1.doc]

**Supplementary information 1** Chemical compositions of Nawametho decoction.

| **Formula** | **Name** | **Mass** | **RT** | **Height** | **Score (DB)** | **Phytochemistry** |
| --- | --- | --- | --- | --- | --- | --- |
| C14 H12 O11 | (+)-Chebulic acid | 356.0386 | 2.092 | 11633000 | 97.73 | tetracarboxylic acids and derivatives |
| C7 H6 O5 | Gallic acid | 170.0216 | 2.865 | 3909346 | 99.93 | gallic acids |
| C10 H12 O7 | 1-O-Galloylglycerol | 244.0584 | 3.069 | 3673793 | 99.61 | galloyl esters |
| C27 H22 O18 | Pterocaryanin B | 634.0806 | 5.17 | 7276249 | 99.52 | hydrolyzable tannins |
| C21 H22 O11 | 2,5,7,4'-Tetrahydroxyflavanone 7-glucoside | 450.1165 | 5.584 | 7459070 | 98.53 | flavanones |
| C21 H22 O10 | (2S)-5,6,7-Trihydroxyflavanone 7-glucoside | 434.1216 | 6.687 | 2196909 | 99.14 | flavanone derivatives |
| C14 H6 O8 | Ellagic acid | 302.0065 | 7.008 | 2060980 | 99.74 | hydrolyzable tannins |
| C27 H30 O15 | Scoparin 2''-O-xyloside | 594.1584 | 7.146 | 2160722 | 99.19 | flavones and flavonols |
| C28 H32 O16 | Rhamnetin 3-robinobioside | 624.1691 | 7.217 | 2345244 | 98.76 | flavones and flavonols |
| C35 H32 O13 | Phylloflavanine | 660.1848 | 7.48 | 4080913 | 98.76 | flavans, flavanols and leucoanthocyanidins |
